# Anti-Inflammatory Effects of Protein Kinase Inhibitor Pyrrol Derivate

**DOI:** 10.1155/2016/2145753

**Published:** 2016-12-22

**Authors:** Halyna M. Kuznietsova, Maryna S. Yena, Iryna P. Kotlyar, Olexandr V. Ogloblya, Volodymyr K. Rybalchenko

**Affiliations:** Institute of Biology, Taras Shevchenko National University, Volodymyrska 64/13, Kyiv 01601, Ukraine

## Abstract

In our previous studies we showed antitumor and anti-inflammatory activities of protein kinases inhibitor pyrrol derivate 1-(4-Cl-benzyl)-3-Cl-4-(CF3-fenylamino)-1H-pyrrol-2,5-dione (MI-1) on rat colon cancer model. Therefore anti-inflammatory effect of MI-1 on rat acetic acid induced ulcerative colitis (UC) model was aimed to be discovered. The anti-inflammatory effects of MI-1 (2.7 mg/kg daily) compared to reference drug Prednisolone (0.7 mg/kg daily) after 14-day usage were evaluated on macro- and light microscopy levels and expressed in 21-grade scale. Redox status of bowel mucosa was also estimated. It was shown that in UC group the grade of total injury (GTI) was equal to 9.6 (GTI_control_ = 0). Increase of malonic dialdehyde (MDA) by 89% and protein carbonyl groups (PCG) by 60% and decrease of superoxide dismutase (SOD) by 40% were also observed. Prednisolone decreased GTI to 3 and leveled SOD activity, but MDA and PCG remained higher than control ones by 52% and 42%, respectively. MI-1 restored colon mucosa integrity and decreased mucosa inflammation down to GTI = 0.5 and leveled PCG and SOD. Thus, MI-1 possessed anti-inflammatory properties, which were more expressed that Prednisolone ones, as well as normalized mucosa redox balance, and so has a prospect for correction of inflammatory processes.

## 1. Introduction

Diseases of the digestive system occupied the second place among all human pathologies after respiratory ones. Inflammatory bowel disease (IBD), which includes ulcerative colitis (UC), is one of the most difficult diseases of the gut for clinical outcome, prevalence, and prognosis. The prevalence of UC in different European countries ranges from 50 to 200 cases per 100 000 population [1]. Moreover, chronic UC has been shown to increase the risk of colorectal cancer up to 8 times [2].

Chronic UC is characterized by erosions and ulcers development in the colon mucosa (in the acute stage), mucosa inflammation, and surface epithelium injury [3]. Very often colon mucosa regeneration is impaired, lipid peroxidation is escalated, and antioxidant system functionality is oppressed [4]. Chronic form of UC in most cases follows the malignant tumor development [5].

The basic aims of chronic UC treatment are to dilute the inflammation and to inhibit the abnormal cell proliferation due to enhanced mucosa regeneration. The primary drugs used in UC traditionally include anti-inflammatory drugs (including ones based on 5-aminosalicylic acid), steroids, and immunosuppressive drugs [6]. However, traditional medications have side effects such as erosion, hemorrhage, and dysfunction of the gut, hepatitis, and pancreatitis, depression of hematopoiesis, and increased risk of infections. Moreover, the risk and severity of them increase with prolonged drugs administration. Furthermore, in some cases the traditional treatment is insufficient to achieve and maintain the remission. So, the need for more potent and more reliable medications is clear.

**Figure 1:**
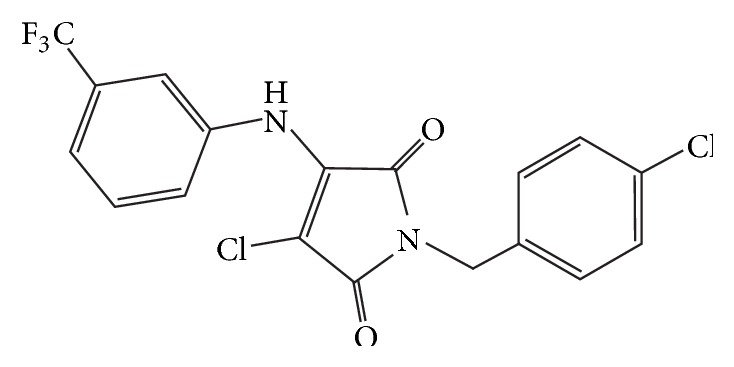
1-(4-Cl-benzyl)-3-Cl-4-(CF_3_-fenylamino)-1Н-pyrrol-2,5-dione (MI-1): structural formula.

Protein kinase inhibitor pyrrol derivate 1-(4-Cl-benzyl)-3-Cl-4-(CF_3_-fenylamino)-1Н-pyrrol-2,5-dione (MI-1) (Figure 1) has been synthesized at Chemistry Department of Taras Shevchenko National University of Kyiv. MI-1 could inhibit such protein kinases as Yes, Src(h), ZAP70, Syk(h), PDK1, EGFR, IGF-1R, VEGFR, and so forth as evidenced by [7, 8]. MI-1 reveals antiproliferative and proapoptotic properties to malignant cells without significant effects on the proliferation and survival of normal ones [7, 9]. Antitumor activity of the agent was also demonstrated on rat model of chemically induced colon cancer [10]. Moreover, MI-1 is relatively safe for the digestive, reproductive, and excretory systems under chronic exposure, as evidenced by [11–13]. Furthermore, when administered to rats experiencing colon cancer, MI-1 attenuates inflammation of mucosa, adjacent to colorectal tumors [10, 11]. MI-1 also reveals antioxidant properties on CoCl_2_-induced oxidative stress model [14].

As MI-1 possesses antitumor and anti-inflammatory activities against experimental colon cancer, as well as antioxidant ones against CoCl_2_-induced oxidative stress; the anti-inflammatory and antioxidant effects of MI-1 on rat UC model were aimed at being discovered. Prednisolone, which is commonly used for moderate UC treatment [6], was chosen as reference drug.

## 2. Materials and Methods

### 2.1. Animals

All experimental procedures executed with animals were in compliance with international principles of the European Convention for the protection of vertebrate animals used for experimental and other scientific purposes (European convention, Strasburg, 1986), article 26 of the Law of Ukraine “On protection of animals from cruelty” (No 3447-IV, 21.02.2006), and all norms of bioethics and biosafety.

40 male Wistar rats weighing 200–220 g (10 weeks old) were obtained from Central Animal House of Taras Shevchenko National University (Kyiv, Ukraine). Five animals were housed per plastic cage on softwood chip bedding and were maintained under constant conditions (12 hr light/dark cycle, 60% humidity at 20–22°C) and fed on standard diet and tap water* ad libitum*.

### 2.2. Chemicals

Ice acetic acid (99.8%, VWR Prolabo, UK) was dissolved immediately before use in distilled water to obtain 4% solution. To induce UC, animals were treated with 1 mL 4% acetic acid solution per rectum weekly for 2 weeks. MI-1 (≥98%, Taras Shevchenko National University of Kyiv) was dissolved in vegetable oil. Animals were treated by MI-1 at the dose of 2.7 mg/kg of body weight* per os* daily. The common anti-inflammatory therapeutic Prednisolone (solution for injections, BioPharma, Ukraine) was used for referencing. Prednisolone was injected intraperitoneally daily at the dose of 0.7 mg/kg of body weight (according to clinical recommendations for moderate UC treatments [6]) in saline.

### 2.3. Experimental Design

Chronic UC was induced as described [15]: animals were induced by 2 rectal applications of 4% acetic acid (1 mL) weekly. After administration, the rats were held upside down for approximately 30 seconds to prevent immediate leakage of the agent from the anus. 15 min before acetic acid applications animals received 3-4 mL of saline intracolonically followed by light belly massage to empty the bowel. MI-1 and Prednisolone applications were started simultaneously with the administration of acetic acid and continued for 2 weeks. Control animals received the appropriate vehicles: saline or vegetable oil.

The rats were divided into 4 groups (10 rats each): (1) vehicle-treated control, (2) UC, the animals were treated with acetic acid, (3) UC + Prednisolone, the animals were treated with acetic acid and with Prednisolone, and (4) UC + MI-1, the animals were treated with acetic acid and with MI-1.

### 2.4. Macroscopic Assay

1 day after the last treatment the rats were sacrificed by carbon dioxide asphyxia, the abdomen was opened, and the entire gastrointestinal tract was removed. The internal bowel surface was visually inspected, the mucosal lesions were scored macroscopically using the following scale: 0, no damage; 1, localized hyperemia and no ulcers; 2, ulceration without hyperemia or bowel wall thickening; 3, ulceration with inflammation at one site; 4, two or more sites of ulceration/inflammation; 5, major sites of damage extending more than 1 cm along the length of colon; 6–10, damage extending more than 2 cm along the length of colon, where the score is increased by one for each additional 1 cm damage [16].

### 2.5. Histological Assay

The 1 cm colons (from 6 to 5 cm proximal to the anus) (distal colon) were fixed for 14 days in neutral saline containing 10% formalin. Then, they were embedded into paraffin and sliced into 5 *μ*m sections, which were stained with hematoxylin-eosin-orange [17] and examined under the light microscope (Olympus BX-41, Olympus Europe GmbH, Japan). The mucosal lesions were scored using 11-grade scale, where total grade was calculated as a sum of grades for bowel mucosa integrity disorders (0–3 grades), leucocytic infiltration (0–3 grades), muscle layer thickening (0–3 grades), crypt abscesses forming (0-1 grade), and goblet cells decrease (0-1 grades) [18]. The grade of total injury (GTI) was also calculated as a sum of grades for mucosa macro- and microscopic lesions.

Mucosa thickness, colonocytes height, and their nuclei cross-sectional area, as well as goblet cells one, were measured from the microphotographs with magnification 400x, using WCIF ImageJ software. The mitotic index (MI) was calculated as the sum of epithelial cells in any phase of mitosis divided on the total sum of epithelial cells. Goblet cells index (GI) and crypt fission index (CFI) were calculated as the sum of goblet cells/fissile crypts divided on the total sum of epithelial cells/crypts.

### 2.6. Biochemical Assays

Bowel internal surfaces were washed with saline followed by PBS containing 1 mM EDTA and 0.4 mM PMSF (serine proteases inhibitor) having рН 7.0. Then mucosa was scraped away and rapidly frozen at −70°C. After being thawed, the samples were gently homogenized in PBS containing 1 mM EDTA and 0.4 mM PMSF and centrifuged at 10000*g* for 15 min, and supernatants were collected and used for analysis. Total protein was estimated quantitatively as described by Lowry et al. [19]. Malonic dialdehyde (MDA), protein carbonyl groups (PCG), intracellular superoxide dismutase (SOD), and catalase (CAT) activities as indicators of colon mucosa redox status were measured spectrophotometrically.

MDA was estimated quantitatively by reaction with thiobarbituric acid as described by [20]. Chromogen absorbance was determined at *λ* = 532 nm at room temperature against blank reference. The extent of lipid peroxidation was expressed as MDA (nmol per mg protein) using a molar extinction coefficient for MDA of 1.56 × 10^5^ M^−1 ^cm^−1^. The level of oxidized proteins (protein carbonyl groups) was estimated quantitatively by oxidized amino acid residues + 2,4-dinitrophenylhydrazine method as described by [21]. Chromogen absorbance was determined at *λ* = 370 nm at room temperature against blank reference. The extent of oxidized proteins was expressed as 2,4-dinitrophenylhydrazone (nmol per mg protein) using a molar extinction coefficient for 2,4-dinitrophenylhydrazone of 2.2 · 10^4^ M^−1 ^cm^−1^. Superoxide dismutase (SOD) activity was quantitatively assessed by nitro blue tetrazolium + riboflavin method as described by [20]. Reaction of O_2_^−^ generation (and nitro blue tetrazolium recovery to formazan) was initiated by bright sunshine for 10 min; formazan absorbance was determined at *λ* = 540 nm at room temperature against blank reference. One unit of SOD activity was considered as 1% inhibition of formazan occurrence. CAT activity was quantitatively assessed by Н_2_О_2_ + molybdenum salt method as described by [22]. Reaction was initiated by adding of 0.03%  Н_2_О_2_ solution to analyzed supernatant and stopped after 10 min by adding of 4% ammonium molybdate solution. Chromogen absorbance was determined at *λ* = 410 nm at room temperature against blank reference. CAT activity was expressed as Н_2_О_2_ splitting (mmol/mg protein per min) using a molar extinction coefficient for Н_2_О_2_ of 22.2 M^−1 ^cm^−1^.

### 2.7. Statistical Analysis

The statistical significance of differences was determined by one-way analysis of variance (ANOVA) with the Bonferroni post hoc test. A value of *p* ≤ 0.05 was considered significant.

## 3. Results

### 3.1. Ulcerative Colitis Group

**Figure 2:**
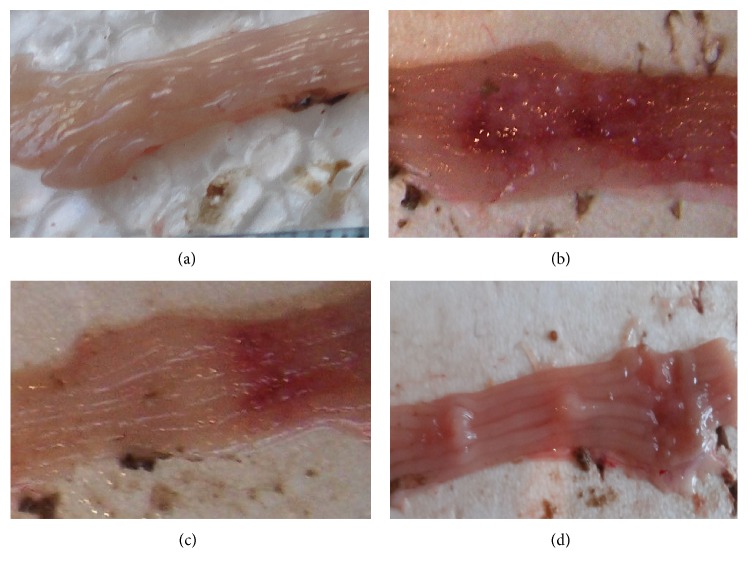
Macrophotographs of bowel internal sides for rats experienced UC and treated with Prednisolone and MI-1. (a) Control group, (b) UC group, (c) UC + Prednisolone group, and (d) UC + MI-1 group.

**Figure 3:**
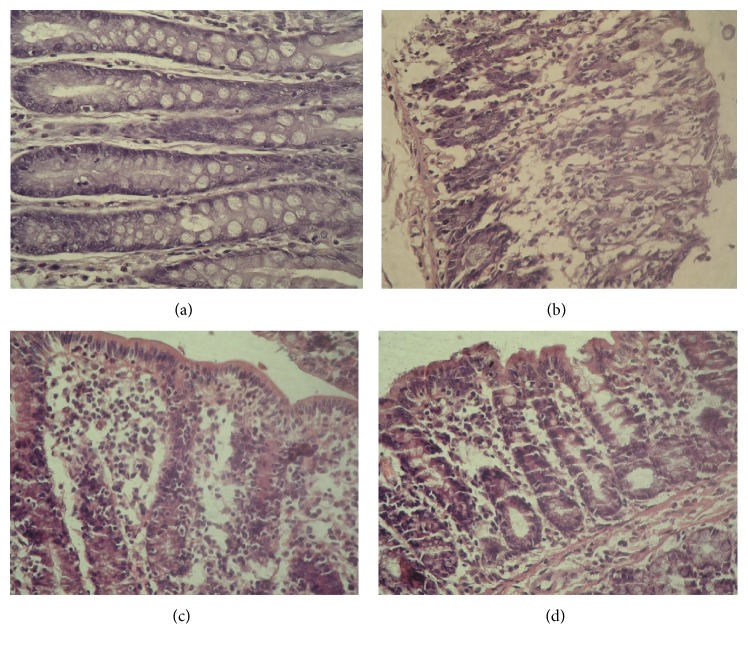
Microphotographs of upper colons for rats experiencing UC and treated with Prednisolone and MI-1. Hematoxylin-eosin orange staining, ×400. (a) Control group, (b) UC group, (c) UC + Prednisolone group, and (d) UC + MI-1 group.

**Figure 4:**
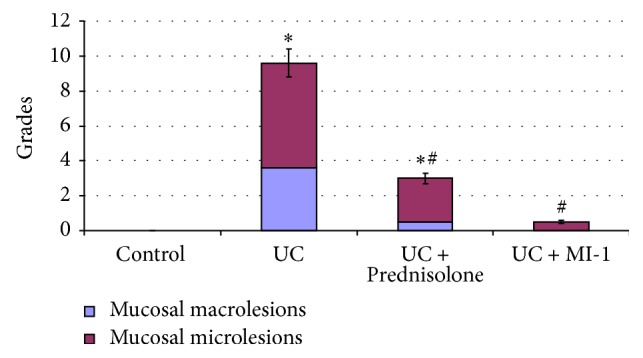
Bowel injury for rats experiencing UC and treated with Prednisolone and MI-1. ^*∗*^*p* ≤ 0.05 compared with control group; ^#^*p* ≤ 0.05 compared with UC group.

Bowel wall thickening, irritation and cornification, adhesions between bowel loops, colonic clefts, and small ulcers on internal bowel surface were observed after visual inspection under UC condition (Figure 2(b)). Edema, epithelium desquamation, fibrinoid, and necrotic layers at areas up to 2 cm along the bowel and inflammatory features manifested by submucosa lymphoid and histiocytic infiltration and extravasation were detected at light microscopy assay (Figure 3(b)). GTI was equal to 9.6 (GTI_control_ = 0) (Figure 4). Mucosa morphometrial parameters in this group were similar to control ones (Table 1), so we concluded [23] the development of chronic UC without atrophy.

Increase of MDA (by 89%) and PCG (by 60%) (Figures 7 and 8) suggested the increase of lipid and protein oxidation and oxidative stress development [24, 25]. Decrease of SOD activity (by 40%) (Figure 6) in bowel mucosa suggested the excess of Н_2_О_2_ or reactive oxygen species [26] and could provoke further escalation of lipid and protein peroxidation and redox imbalance. Also CAT activity tended to decrease compared to control one (Figure 6). These observations are typical for inflammatory processes [25] and confirm the UC development.

### 3.2. UC + Prednisolone Group

In this experimental group no bowel lesions, except light edema, were observed under visual inspection (Figure 2(c)), as well as no ulcers, edema, and epithelium desquamation at light microscopy analysis. Nevertheless, some inflammatory features (submucosa lymphoid and histiocytic infiltration) persisted (Figure 3(c)); thus GTI was equal to 3 (Figure 4). Mucosa morphometrial parameters in this group were similar to control ones (Table 1), but GI was less than UC one by 20%, suggesting some inhibition of mucous formation and/or secretion.

**Figure 5:**
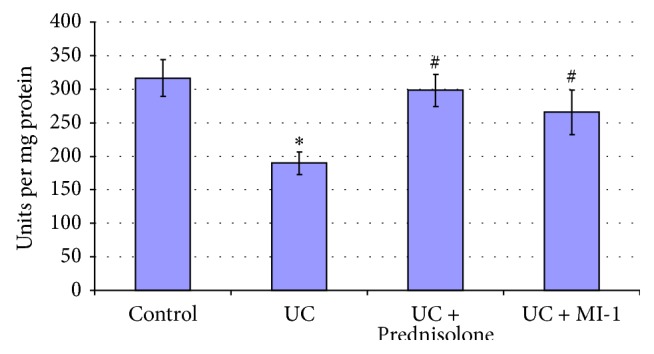
Bowel mucosa SOD activity for rats experiencing UC and treated with Prednisolone and MI-1. ^*∗*^*p* ≤ 0.05 compared with control group; ^#^*p* ≤ 0.05 compared with UC group.

**Figure 6:**
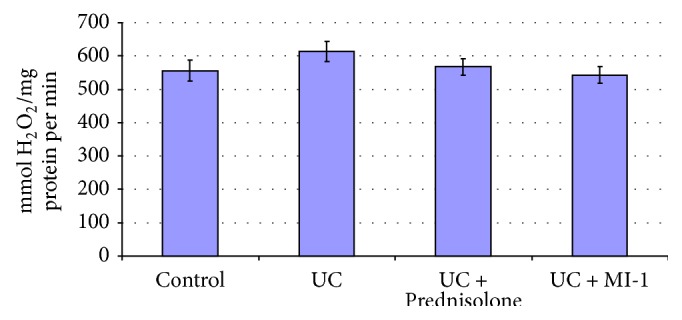
Bowel mucosa CAT activity for rats experiencing UC and treated with Prednisolone and MI-1.

**Figure 7:**
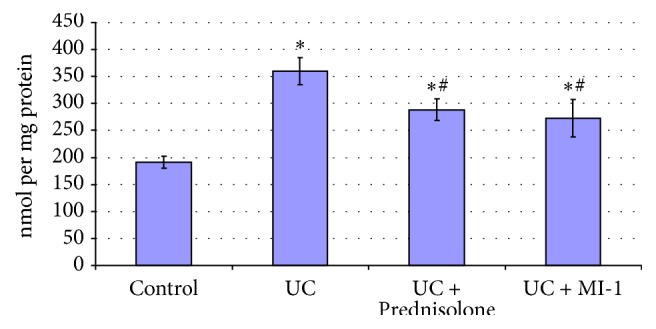
Bowel mucosa MDA concentrations for rats experiencing UC and treated with Prednisolone and MI-1. ^*∗*^*p* ≤ 0.05 compared with control group; ^#^*p* ≤ 0.05 compared with UC group.

**Figure 8:**
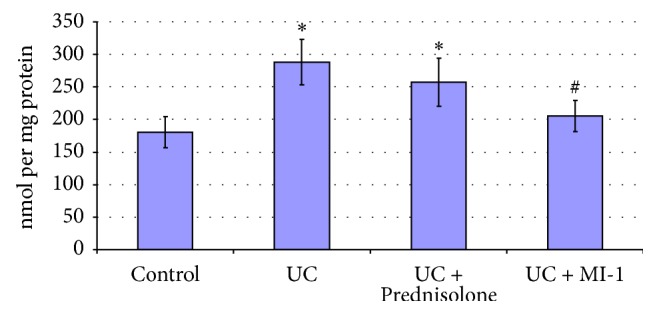
Bowel mucosa PCG concentrations for rats experiencing UC and treated with Prednisolone and MI-1. ^*∗*^*p* ≤ 0.05 compared with control group; ^#^*p* ≤ 0.05 compared with UC group.

SOD activity was leveled by Prednisolone (Figure 5), but MDA and PCG remained higher than control ones by 51% and 42%, respectively, despite their decrease (by 20% and 11%, resp.) compared with UC group (Figures 7 and 8). These findings suggested the attenuation of inflammation and oxidative stress level by Prednisolone, as well as partial restore of mucosa integrity.

### 3.3. UC + MI-1 Group

No bowel lesions after visual inspection (Figure 2(d)) and no ulcers, edema, and epithelium desquamation at microscopic level were observed at this group. Moreover, mucosa inflammation decreased down to local foci of lymphocytes and histiocytes (Figure 3(d)); thus GTI was equal to 0.5 (Figure 4). Mucosa morphometrial parameters in this group were similar to control ones (Table 1), but colonocytes' nuclei area exceeded the UC one by 23%, suggesting the activation of colonocytes' functional state. Also CFI decreased by 54% compared to UC and tended to decrease by 44% compared to control ones.

MI-1 also leveled PCG and SOD (Figures 5 and 8), but MDA remained higher than control one by 43%, despite its decrease (by 24%) compared with UC group (Figure 7). So MI-1 more effectively restored colon mucosa integrity and attenuated inflammation and oxidative stress level than Prednisolone did.

## 4. Discussion

The mechanisms of anti-inflammatory action of glucocorticoids are well established. Thus, glucocorticoids are capable of suppressing the inflammatory process through altering the expression of corticosteroid-responsive genes. Glucocorticoid-specific receptors in the cell cytoplasm bind with steroid ligands to form hormone-receptor complexes that eventually translocate to the cell nucleus. There, these complexes bind to specific DNA sequences and alter their expression. The complexes may induce the transcription of mRNA, leading to synthesis of new proteins. Such proteins include lipocortin, a protein known to inhibit phospholipase A2 and thereby block the synthesis of prostaglandins and leukotrienes from arachidonic acid. Glucocorticoids also inhibit the production of other mediators, including arachidonic acid metabolites such as those produced via cyclooxygenase (COX) activation (both COX-1 and COX-2), cytokines, interleukins, adhesion molecules, and enzymes such as collagenase. Hormone-receptor complex also has the ability to repress the activity of NF-*κ*B, one of the main regulators of inflammatory response, directly [27, 28].

The possible mechanisms of action of tyrosine kinase inhibitors are not so clear. MI-1 was synthesized as protein kinases inhibitor [8]. It was tested on 32 protein kinases for determination of inhibitory profile and has been shown to inhibit EGF-R, FGF-R1, IGF1-R, INS-R, VEGF-R1, VEGF-R2, VEGF-R3, Syk, ZAP70, PDK1, Yes, and Src(h) [7]. It is known that EGF-R, VEGFR, IGF1R, Src, and PDK1 are overexpressed in human colorectal tumors [29, 30], so the mechanism of MI-1 antitumour action could consist on inhibition of these kinases in tumor tissue. But it is also known that some of these receptors and proteins are involved in inflammatory process. Thus, EGFR and IGF1R, which are the targets of MI-1, act as the major upstream activators of PI3K/Akt pathway. Moreover, PDK1, another target of MI-1, is the key effector of this pathway. Activation of PI3K/Akt pathway leads to activation of NF-*κ*B [31], which regulates host inflammatory and immune responses, as well as cellular growth. Activation of NF-*κ*B pathway is involved in the pathogenesis of chronic inflammatory diseases, such as asthma, rheumatoid arthritis, and IBD. Inhibition of NF-*κ*B pathway by glucocorticoids and nonsteroidal anti-inflammatory drugs is associated with anti-inflammatory properties of these medications [32]. Thus, anti-inflammatory properties of EGFR-, IGF1R-, and PDK1-inhibitors could be explained by inhibition of PI3K/Akt/NF-*κ*B signaling pathway [33].

Recent publications suggest that altered angiogenesis may be a critical component of IBD pathogenesis. Angiogenesis in the area of chronic inflammation, such as that observed in IBD, is abnormal and characterized by distorted vasculature, increased permeability, and thrombogenic potential. Moreover, abnormal angiogenesis may facilitate migration of inflammatory cells to the site of inflammation, leading to the perpetuation of chronic inflammation. Indeed, angiogenesis is intrinsic to chronic inflammation and is associated with structural changes, including activation and proliferation of endothelial cells and capillary and venule remodeling, all of which result in expansion of the tissue microvascular bed [34]. A potential functional consequence of this expansion is the promotion of inflammation through increase of inflammatory cells influx and nutrient supply to the metabolically active immune process and the local production of cytokines, chemokines, and matrix metalloproteinases by activated endothelium [35]. Thus, angiogenesis and inflammation become chronically codependent processes. It has now been clearly established that the microvascular changes associated with angiogenesis are key contributors to the tissue injury and remodeling process that accompanies chronic inflammation. VEGF is the predominant regulator of angiogenesis through the initiation of vessel growth, the inhibition of endothelial cell apoptosis, and the incorporation of hematopoietic and endothelial progenitor cells into the developing vasculature. Because growing evidence supports a role for angiogenesis in chronic inflammation [36], the inhibition of VEGF signaling has also been proposed as a promising therapeutic strategy in this disorder. In fact, it was shown that the inhibition of VEGF signaling in dextran sodium sulfate-induced colitis reduces inflammation, whereas the overexpression of VEGF leads to an augmentation of intestinal inflammation [37]. It was also evidenced [38] that in inflamed tissue VEGF is highly expressed not only in endothelial cells, but also in leukocytes and epithelial cells. Treatment with anti-VEGF antibody markedly improves the clinical and morphologic features of UC in part by reducing excessive vascular permeability and decreasing inflammatory cells infiltration. Therefore, anti-inflammatory effects of MI-1 could be mediated by VEGFR pathway blockade.

Syk is a key protein of the immunoreceptor signaling pathway in immune and inflammatory responses. Syk has been proposed to play a critical role in psoriasis, atherosclerosis, and IBD. The data [39] showed that Syk expression increases in experimental colitis, and fostamatinib, a Syk inhibitor, affects inflammatory cells and proinflammatory cytokines and provides histological and morphological healing of colon mucosa. MI-1 could inhibit Syk in vitro, so its anti-inflammatory effects could be realized through signaling pathways which involve Syk.

Oxidative stress, among the immune-regulatory factors, has been proposed as one of the major mechanisms involved in the pathophysiology of IBD. The predominant source of reactive oxygen species (ROS) in the gut mucosa is the NADPH oxidases (NOX). NOX 1 is highly expressed in colon epithelial cells, where it generates ROS to interact with normal and pathogenic bacteria. Excessive reactive ROS production is associated with damage to the intestinal mucosa at IBD. Moreover, chronic inflammation in IBD is characterized by massive leukocyte infiltration of the gut. On activation, these cells produce not only a wide spectrum of proinflammatory cytokines but also an excessive amount of ROS, which markedly alters the redox equilibrium within the gut mucosa, and maintain inflammation by inducing redox-sensitive signaling pathways and transcription factors. Recent developments point to the possibility of treating the oxidative stress associated with IBD by using chemical compounds with antioxidant potential [4]. In our study we showed that MI-1 decreases the formation of lipid and protein peroxides and normalizes the attenuated SOD activity and so acts like an antioxidant. In our previous studies antioxidant properties of MI-1 were also observed [40]. Thereby we propose that MI-1 could impact tissue redox equilibrium through inhibition of proinflammatory signaling pathways (e.g., PI3K/Akt/NF-*κ*B).

It was shown that activation of EGFR is associated with ROS production, which transiently inactivates protein tyrosine phosphatases to enhance or prolong EGFR activation [6]. Moreover, abnormalities in EGFR activation in the context of chronic diseases such as cancer or IBD may not only simply be due to altered expression of EGFR or its ligands but also be related to altered expression or activation of NOX enzymes that regulate this signaling pathway [41]. Also exogenous ROS stimulates the induction of VEGF expression in endothelial cells, smooth muscle cells, and macrophages. At the same time, VEGF further stimulates ROS production through the activation of NOX in endothelial cells. In several pathologies, exemplified by diabetic retinopathy and injured arteries, ROS-mediated angiogenesis is strongly associated with VEGF expression [42]. So inhibition of EGFR and VEGFR could be associated with decreased ROS production. Therefore we propose that MI-1 could inhibit ROS production and normalize impaired redox balance through inhibition of EGFR and VEGFR.

Indeed, protein kinases are strongly involved in pathogenesis of IBD and thereby could be the targets of anti-IBD therapy. For example, tofacitinib which is a small molecular inhibitor of the Janus kinase is currently approved for the treatment of rheumatoid arthritis and recently has been shown to be effective in the treatment of UC [43]. So the therapeutic potential of targeted protein kinase inhibitors including pyrrol derivate MI-1 is extended.

## 5. Conclusions

MI-1 possessed anti-inflammatory properties more expressed than Prednisolone ones, as well as normalized mucosa redox equilibrium. Since anti-inflammatory effects of glucocorticoids are realized through inhibition of phospholipase A2 and COX-2, whereas effects of MI-1 through inflammatory and ROS signaling pathways are multiple, probably determining more expressed and complex impact of MI-1 on inflammatory process. Thus MI-1 has a prospect for correction of inflammatory process through multiple mechanisms of action.

## Figures and Tables

**Table 1:** Morphometric parameters of colon mucosa for rats experiencing UC and treated with Prednisolone and MI-1. Mean ± SD, *n* = 10^*∗*^.

	Control	UC	UC + Prednisolone	UC + MI-1
Mucosa thickness, *μ*m	588.5 ± 93.8	648.7 ± 103.6	651.5 ± 129.4	667.5 ± 133.6
Colonocytes height, *μ*m	17.2 ± 3.6	17.4 ± 2.6	16.4 ± 2.4	16.0 ± 2.2
Colonocytes nuclei cross-sectional area, *μ*m^2^	22.7 ± 4.8	22.0 ± 4.2	23.9 ± 1.6	27.0 ± 4.4 (*p* = 0.04)
Goblet cells cross-sectional area, *μ*m^2^	82.1 ± 10.2	69.4 ± 18.0	65.2 ± 14.0	71.9 ± 8.4
GI, %	26.7 ± 3.2	28.8 ± 1.4	23.0 ± 3.0 (*p* = 0.008)	27.5 ± 3.8
MI, %	5.2 ± 0.8	5.0 ± 1.4	4.6 ± 1.2	5.1 ± 0.6
CFI, %	5.7 ± 2.6	7.0 ± 1.2	6.4 ± 1.4	3.2 ± 1.0 (*p* = 0.007)

^*∗*^ *p* values indicated in comparison with UC group.

## Abbreviation List

IBD: Inflammatory bowel disease
UC: Ulcerative colitis
MI-1: 1-(4-Cl-benzyl)-3-Cl-4-(CF3-fenylamino)-1H-pyrrol-2,5-dione
Yes: Yamaguchi sarcoma viral oncogene homolog 1
Src(h): Rous sarcoma oncogene cellular homolog
ZAP70: Zeta-chain-associated protein kinase 70
Syk(h): Spleen tyrosine kinase
PDK1: 3-Phosphoinositide-dependent kinase 1
EGFR: Endothelial growth factor receptor
IGF1R: Insulin-like growth factor-1 receptor
VEGFR: Vasoendothelial growth factor receptor
GTI: Grade of total injury
MI: Mitotic index
GI: Goblet cells index
CFI: Crypt fission index
PBS: Phosphate-buffered saline
EDTA: Ethylenediaminetetraacetic acid
PMSF: Phenylmethylsulphonyl fluoride
MDA: Malonic dialdehyde
PCG: Protein carbonyl groups
SOD: Superoxide dismutase
CAT: Catalase
COX: Cyclooxygenase
INS-R: Insulin receptor
PI3K/Akt: Phosphatidylinositol-3 kinase/protein kinase B
NF-*κ*B: Nuclear factor *κ*B
ROS: Reactive oxygen species
NOX: Nicotinamide adenine dinucleotide phosphate oxidase.
